# Menthol Stereoisomers Exhibit Different Effects on α4β2 nAChR Upregulation and Dopamine Neuron Spontaneous Firing

**DOI:** 10.1523/ENEURO.0465-18.2018

**Published:** 2019-01-04

**Authors:** Brandon J. Henderson, Stephen Grant, Betty W. Chu, Rezvan Shahoei, Stephanie M. Huard, Shyam S. M. Saladi, Emad Tajkhorshid, Dennis A. Dougherty, Henry A. Lester

**Affiliations:** 1 Division of Biology and Biological Engineering, California Institute of Technology, Pasadena, California 91125; 2 Department of Biomedical Sciences, Joan C. Edwards School of Medicine at Marshall University, Huntington, West Virginia 25703; 3 Division of Chemistry and Chemical Engineering, California Institute of Technology, Pasadena, California 91125; 4 Department of Physics, National Institutes of Health Center for Macromolecular Modeling and Bioinformatics, Beckman Institute for Advanced Science and Technology, University of Illinois at Urbana-Champaign, Urbana, Illinois 61801; 5 Department of Biochemistry, National Institutes of Health Center for Macromolecular Modeling and Bioinformatics, Beckman Institute for Advanced Science and Technology, Center for Biophysics and Quantitative Biology, University of Illinois at Urbana-Champaign, Urbana, Illinois 61801

**Keywords:** cigarettes, electronic nicotine delivery systems, nicotine, nicotine addiction, *Xenopus* oocyte

## Abstract

Menthol contributes to poor cessation rates among smokers, in part because menthol enhances nicotine reward and reinforcement. Mentholated tobacco products contain (−)-menthol and (+)-menthol, in varying proportions. We examined these two menthol stereoisomers for their ability to upregulate α4β2 nAChRs and to alter dopamine neuron firing frequency using long-term, low-dose (≤500 nm) exposure that is pharmacologically relevant to smoking. We found that (−)-menthol upregulates α4β2 nAChRs while (+)-menthol does not. We also found that (−)-menthol decreases dopamine neuron baseline firing and dopamine neuron excitability, while (+)-menthol exhibits no effect. We then examined both stereoisomers for their ability to inhibit α4β2 nAChR function at higher concentrations (>10 µm) using the *Xenopus* oocyte expression system. To probe for the potential binding site of menthol, we conducted flooding simulations and site-directed mutagenesis. We found that menthol likely binds to the 9´ position on the TM2 (transmembrane M2) helix. We found that menthol inhibition is dependent on the end-to-end distance of the side chain at the 9´ residue. Additionally, we have found that (−)-menthol is only modestly (∼25%) more potent than (+)-menthol at inhibiting wild-type α4β2 nAChRs and a series of L9´ mutant nAChRs. These data reveal that menthol exhibits a stereoselective effect on nAChRs and that the stereochemical effect is much greater for long-term, submicromolar exposure in mice than for acute, higher-level exposure. We hypothesize that of the two menthol stereoisomers, only (−)-menthol plays a role in enhancing nicotine reward through nAChRs on dopamine neurons.

## Significance Statement

Menthol is the most popular flavor used in all forms of tobacco products. Depending on the method used to produce the menthol, these products contain two stereoisomers [(−)-menthol and (+)-menthol] in unregulated and poorly characterized amounts. We studied how these isomers of menthol differ in their ability to alter nicotinic receptors on dopamine neurons that contribute to tobacco addiction. Long-term treatment with (−)-menthol and (+)-menthol resulted in a stereospecific effect on nicotinic receptor upregulation and dopamine neuron excitability: (+)-menthol exhibited no effect while (−)-menthol exhibited a robust effect. In acute treatments, we found only slight differences between the effects of the two isomers on nicotine receptors. This suggests that long-term exposure to (−)-menthol may contribute to exacerbating tobacco addiction.

## Introduction

Menthol cigarettes are used by a third of smokers and by the majority of African-American smokers (McCarthy et al., 1995). Smokers of menthol cigarettes are less likely to quit when compared with smokers of nonmenthol cigarettes (Ahijevych and Garrett, 2010). Youth smokers of menthol cigarettes are twice as likely to become lifelong smokers compared with youth smokers of nonmenthol cigarettes (D'Silva et al., 2012). Menthol is present in much of the electronic nicotine delivery system (ENDS) market (Centers for Disease Control and Prevention, 2016). Some countries, as well as some municipalities in the United States, ban menthol in combustible tobacco products. Apart from these exceptions, menthol holds a unique position as a tobacco flavoring: it is the only flavor allowed in combustible cigarettes in the United States following the 2009 Family Smoking Prevention and Tobacco Control Act.

Recent investigations of menthol identified pharmacological effects on several members of the Cys-loop family of receptors: menthol is (1) a negative allosteric modulator of α4β2 nAChRs, (2) a noncompetitive antagonist of α7 and α3β4 nAChRs, (3) a noncompetitive antagonist of 5-HT_3_ receptors, (4) a positive allosteric modulator of GABA_A_ receptors, and (5) a positive modulator of glycine receptors (Hall et al., 2004; Hans et al., 2012; Ashoor et al., 2013a, b; Lau and Vaughan, 2014; Ton et al., 2015). While most investigations examined (−)-menthol only, one identified a difference in the actions of (+)-menthol and (−)-menthol (Hall et al., 2004). Hall et al. (2004) observed that (+)-menthol is a potent allosteric potentiator of GABA_A_-mediated currents, while (−)-menthol provided a modest enhancement.

Eight stereoisomers of menthol exist. Plants produce mainly the stereoisomer [1*R*, 2S, 5*R* or (−)-menthol; Chen et al., 2011); but small quantities of additional stereoisomers occur in various types of mint leaves or in the extracted oil). As with many popular natural products, demand for menthol greatly exceeds the supply that can be obtained from plants. In the past, the primary synthetic method for producing menthol used hydrogenation of pulegone, producing menthol as a racemate [(+)-menthol and (−)-menthol; Sell, 2006]. However, Ohkuma et al. (2000) developed an asymmetric synthesis method that provides pure (−)-menthol. Thus, while (±)-menthol and (+)-menthol are easily produced synthetically and have been found in menthol tobacco products (Heck, 2010), (−)-menthol has now become the primary stereoisomer in tobacco products (Chen et al., 2011).

Previous data show that menthol by itself upregulates nAChRs (Alsharari et al., 2015; Henderson et al., 2016) and alters midbrain dopamine (DA) neuron firing (Henderson et al., 2016). Because both (+)-menthol and (−)-menthol may be present in menthol cigarettes, we investigated the potential differences that these two stereoisomers may exhibit on nAChR upregulation and dopamine neuron firing. We show that only (−)-menthol is responsible for the upregulated α4β2 nAChRs and decreased dopamine neuron firing frequency found when neurons are treated with (±)-menthol. We also found that (+)-menthol is 25% less potent at inhibiting α4β2 nAChRs. The concentrations of menthol that inhibit α4β2 nAChRs are similar to the concentrations required to activate the TRPM8 (transient receptor potential cation channel subfamily M member 8) channel, which is mainly responsible for the cooling sensation elicited by menthol (Ha et al., 2015). We also investigated a putative binding site for menthol on α4β2 nAChRs using computational modeling and site-directed mutagenesis. Here we determined that menthol likely binds to the 9´ site of the transmembrane M2 (TM2) helix of α4 nAChR subunits, and its binding interaction depends on steric interactions. Together, these data show that menthol exhibits different actions when applied in the long term or acutely. Long-term exposure to menthol exhibits a stereospecific effect on nAChR upregulation and dopamine neuron excitability: (−)-menthol produces a robust effect, and (+)-menthol seems to be inert. This suggests that (−)-menthol, and not (+)-menthol, possesses the potential risk of enhancing nicotine reward and reinforcement.

## Materials and Methods

### Reagents

(+)-Menthol (product number 63658), (−)-menthol (product number M2780), and ACh chloride (product number A6625) were obtained from Sigma-Aldrich.

### Mice and cultured midbrain neurons

In electrophysiological experiments, we used pregnant female Gene Expression Nervous System Atlas tyrosine hydroxylase (TH)-eGFP mice (Henderson et al., 2016, 2017) that were purchased from the Mutant Mouse Regional Resource Center (Chapel Hill, NC). All experiments were conducted in accordance with the guidelines for the care and use of animals provided by the National Institutes of Health, and protocols were approved by the Institutional Animal Care and Use Committee at the California Institute of Technology. To culture primary midbrain neurons from the TH-eGFP mice, we used embryonic day 14 embryos. We did not determine the sex of the embryos; but, given the mixed population of males and females in litters, the resulting midbrain cultures were composed of neurons from both sexes. (without determining their sex). Ventral midbrain tissue was dissected and cultured using previously published procedures (Srinivasan et al., 2016). Following digestion with papain (15 min at 37°C), cells were separated by DNase treatment and trituration, and were plated at a density of 100,000 cells/dish in poly-l-ornithine- and laminin-coated imaging dishes containing Neurobasal medium supplemented with B27, Glutamax, 1% hyclone equine serum, and 100 µm ascorbate. For drug treatments, control medium (control), 500 nm (−)-menthol, or 500 nm (+)-menthol were added to the cultures for 10 d. Culture medium was changed at ∼3 d intervals.

### Neuro-2a cell culture and transient transfections

Neuro-2a cells were cultured using standard techniques (Srinivasan et al., 2011). For imaging, cells were plated by adding 90,000 cells to poly-d-lysine-coated 35 mm glass-bottom imaging dishes (MatTek) and cultured in a humidified incubator (37°C, 95% air, 5% CO_2)_. Cells were transfected as described previously (Henderson et al., 2014). Similar to previous assays (Henderson et al., 2016), drug treatments (control or 500 nm menthol stereoisomers) were applied for 24 h. Culture medium containing menthol was removed 1 h before the total internal reflection fluorescence microscopy (TIRFM) assays and replaced with extracellular solution (ECS), identical to methods previously described (Henderson et al., 2016, 2017).

### Total internal reflection fluorescence microscopy

TIRFM enables the visualization of fluorescently labeled intracellular molecules on or near the plasma membrane (within ∼250 nm of the cell–coverslip interface). Cultured neuro-2a cells were imaged live at 37°C in a stage-mounted culture dish incubator (Warner Instruments) using methods and instrumentation reported previously (Richards et al., 2011; Henderson et al., 2014). For each TIRFM session, image analysis was started 25 h after the beginning of drug treatment (24 h for drug treatment, 1 h for washout with ECS). This ensured that each session recorded cells at the same time point. For each session and each drug condition, 30–40 cells were imaged. Each condition was measured in at least three separate imaging sessions.

### Patch-clamp electrophysiology

Neurons were visualized with an upright microscope (BX50WI, Olympus) using near-infrared or blue illumination (the latter for visualizing GFP fluorescence). Whole-cell patch-clamp techniques were used to record electrophysiological signals. Data were sampled at 10 kHz and filtered at 2 kHz. Patch electrodes had a resistance of 4–8 MΩ, when filled with intracellular solution (in mm: 135 K gluconate, 5 KCl, 5 EGTA, 0.5 CaCl_2_, 10 HEPES, 2 Mg-ATP, and 0.1 GTP). The ECS was composed as follows (in mm): 140 NaCl, 5 KCl, 2 CaCl_2_, 1 MgCl_2,_ 10 HEPES, and 10 glucose. The pH of these solutions was adjusted to 7.2 with Tris-base, and their osmolarity was adjusted to 300 mOsm with sucrose.

All recordings were performed at a mean temperature of 32 ± 1°C. ACh (300 µm) was puffed onto neurons using a Picospritzer III for a duration of 300 ms (holding levels: voltage clamp, −65 mV; current clamp, 0 pA). Neurons were selected by the presence or absence of TH-eGFP fluorescence and labeled as putative DA or GABA neurons. Firing frequency and action potential duration were recorded (>1 min) to further verify GFP or non-GFP distinctions of DA or GABA neurons. Drug treatments for cultured midbrain neurons were performed for 10 d (control or 500 nm either menthol stereoisomer; as described above). At the beginning of a recording session, dishes containing cultured midbrain neurons were rinsed with ECS to remove any remaining drug treatment. Cultured neurons were placed on the recording stage and perfused with ECS for 1 h to ensure that all remaining menthol from chronic treatments was removed. For all recording sessions, perfusion rates were maintained at constant flow so that changes in agonist washout did not complicate data interpretation.

### Oocyte preparation and injection

Rat α4 and β2 nAChR subunits were in pGEMhe vectors. The mRNAs were prepared from linearized DNA, using a T7 mMessage mMachine kit (Ambion) and were purified with the RNeasy Mini Kit (Qiagen). *Xenopus laevis* stage V and VI oocytes were harvested via standard protocols (Marotta et al., 2014). The α4 and β2 mRNAs were mixed in a 1:10 ratio by mass to obtain the (α4)_2_(β2)_3_ or in a 10:1 ratio to obtain the (α4)_3_(β2)_2_ receptor. For unnatural amino acid incorporation, a TAG codon was incorporated into the site of interest, and 40 ng of unnatural amino acid-coupled THG73 tRNA was added to the injection solution. The α4 and β2 mRNAs were mixed in a 100:1 ratio by mass to obtain the (α4)_3_(β2)_2_ receptor for these unnatural amino acid experiments. A total of 50 nl of the RNA mixture were injected into each oocyte, delivering an mRNA mass total of 22 ng. After injection, the oocytes were incubated at 18°C in ND96 medium (see below) enriched with theophylline, sodium pyruvate, and gentamicin for 48 h before recording.

### Oocyte electrophysiology

The OpusXpress 6000A (Molecular Devices) in two-electrode voltage-clamp mode was used for all electrophysiological recordings. ACh was dissolved to 1 m stock solutions in ND96 Ca^2+^-free buffer (96 mm NaCl, 2 mm KCl, 1 mm MgCl_2_, and 5 mm HEPES at pH 7.5). The holding potential was set to −60 mV, and the running buffer used was ND96 Ca^2+^-free solution for all experiments. All drugs were applied as a 1 ml application in ND96 Ca^2+^ buffer. Drug applications used 1 ml of drug solution applied over 15 s followed by a 5 min buffer wash at a rate of 3 ml/min. Data were sampled at 50 Hz and then low-pass filtered at 5 Hz. Averaged and normalized data were fit to one Hill term to generate EC_50_, IC_50_, and Hill coefficient (n_H_) values. All currents for the activity testing were normalized to the maximum current produced by the oocyte for a given compound (I_max_) from ACh potentiation. The efficacy of compounds was measured as the ratio of the I_max_ of the compound divided by the I_max_ of the ACh. Error bars represent SEM values.

### Molecular dynamics simulations

To characterize putative binding sites of menthol to the human α4β2 nicotinic receptor, flooding simulations were performed using NAMD 2.12 (Phillips et al., 2005). Flooding simulations are molecular dynamics (MD) simulations in which many identical molecules are initially placed in solution and allowed to diffuse and interact with the various environments such as water, membrane, and proteins.

For the protein structure, we used the x-ray crystal structure of the human α4β2 nicotinic receptor (Morales-Perez et al., 2016; Protein Data Bank code, 5KXI). To prepare the protein for the simulation, we first used ModWeb (Sánchez and Sali, 1998), an automated comparative protein structure modeling web server, to build the missing intracellular loops (which connect M3 and M4 helices) for both α4 and β2 subunits. Then, CHARMM-GUI (Jo et al., 2008) was used to embed the protein into a 120 × 120 Å POPC lipid bilayer in the x–y-plane. The x-ray crystal structure captures the receptor in its desensitized state with two nicotine molecules bound to the extracellular domain of the receptor. In our simulation, we did not include the two bound nicotine molecules. To preserve the crystallographically captured state of the protein, we imposed harmonic restraints on backbone atoms over the course of the simulation. The SOLVATE and AUTOIONIZE plugins in VMD (Humphrey et al., 1996) were used to solvate and to ionize the system to an NaCl concentration of 150 mm, respectively.

We started the flooding simulation with 178 menthol molecules (corresponding to a concentration of ∼165 mm) randomly placed in the solution. In our preliminary simulations, the majority of menthol molecules aggregated in the solution before getting a chance to interact with the lipid head groups or the protein, an expected outcome given the highly hydrophobic nature of menthol (logD = 2.7). To overcome this problem, nonbonded repulsive energy terms were introduced between the two oxygen atoms of each pair of menthol molecules (using NBFix corrections). We chose to decrease the well depth of the Lennard–Jones potential to −0.10 kcal/mol instead of its original value of −0.1921 kcal/mol. We also increased the minimum interparticle distance between each pair of oxygen atoms from two menthol molecules from 3.53 to 12.00 Å. As a result, pairs of menthol molecules were prevented from aggregating. Given the NAMD cutoff of 12 Å for nonbonded interactions, by using these NBFix corrections, we basically turned off the attraction between any two oxygen atoms that belong to separate menthol molecules. The production run was performed for 800 ns. After the first 350 ns, the majority of menthol molecules partitioned into the membrane, allowing us to turn off the NBFix corrections so that multiple menthol molecules were not prevented from approaching the same binding site/region on the protein.

The simulations were performed under periodic boundary conditions. Long-range electrostatic interactions were calculated by the PME (Particle mesh Ewald method; Darden et al., 1993; Essmann et al., 1995) with one grid point density per angstrom. Except for the very first phase of melting the lipid tails, where the NVT (constant temperature, constant volume) ensemble is preferred, the NPT (constant temperature, constant pressure) ensemble was used. The constant temperature of 310 K for the system was provided by coupling it to a heat bath and through Langevin dynamics for all nonhydrogen atoms with a Langevin damping coefficient of 5 ps^−1^. For the NPT simulation, the pressure was kept constant at 1 atmosphere through coupling to a Nosé-Hoover Langevin piston, with a period of 200 fs and a damping timescale of 50 fs. Time steps of 2 fs were chosen for the simulation. For the water molecules, the TIP3P model (Jorgensen et al., 1983) was used. CHARMM36 (Klauda et al., 2010) force field parameters were used for the protein, ions, and lipids. For menthol, we used the CHARMM General Force Field (CGenff; Vanommeslaeghe and MacKerell, 2012; Yu et al., 2012) charges and parameters, calculated by the CGenff web interface program (Vanommeslaeghe et al., 2010, 2012).

### Code and software availability

NAMD 2.12 was used for all of the molecular dynamics simulations, and VMD was used for visualization and analysis of the simulation results. Both of these software packages are free and available for all users at https://www.ks.uiuc.edu/Research/namd/ and at https://www.ks.uiuc.edu/Research/vmd/, respectively.

### Experimental design and statistical analysis

**Figure 1.**
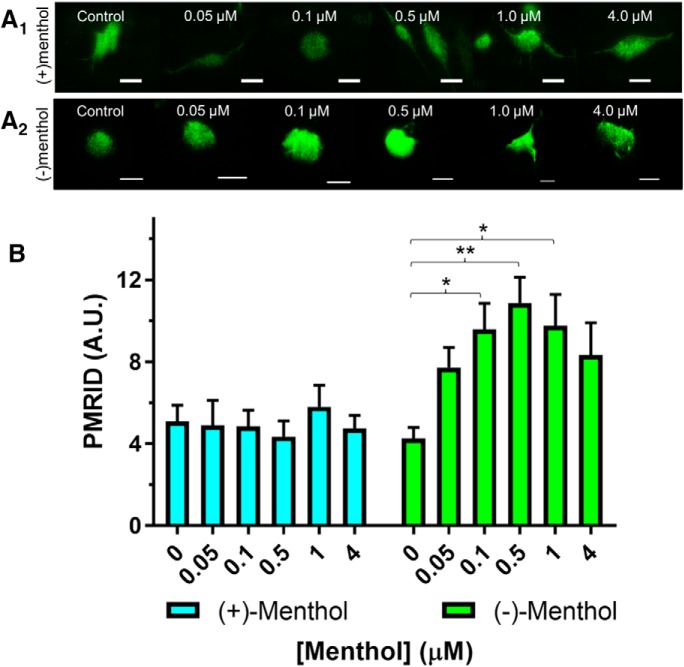
Chronic exposure of menthol stereoisomers causes different effects on α4β2 nAChR upregulation. ***A***, Representative TIRFM images of neuro-2a cells transfected with α4-SEP and β2 nAChR subunits. Menthol stereoisomers (500 nm) were added 24 h before imaging sessions. Scale bars, 10 µm. Each panel image shows representative cells at pH 7.4. ***B***, PMRID was quantified for SEP nAChRs following treatment with menthol stereoisomers. For each condition, *n* > 30 cells. Data are the mean ± SEM. *p < 0.05; **p < 0.01 (one-way ANOVA with Tukey). Two-way ANOVA, (+)-menthol vs (−)-menthol, *F*_(1,381)_ = 30.34 and *p* < 0.0001. Exact *p* values are provided in the text.

**Figure 2.**
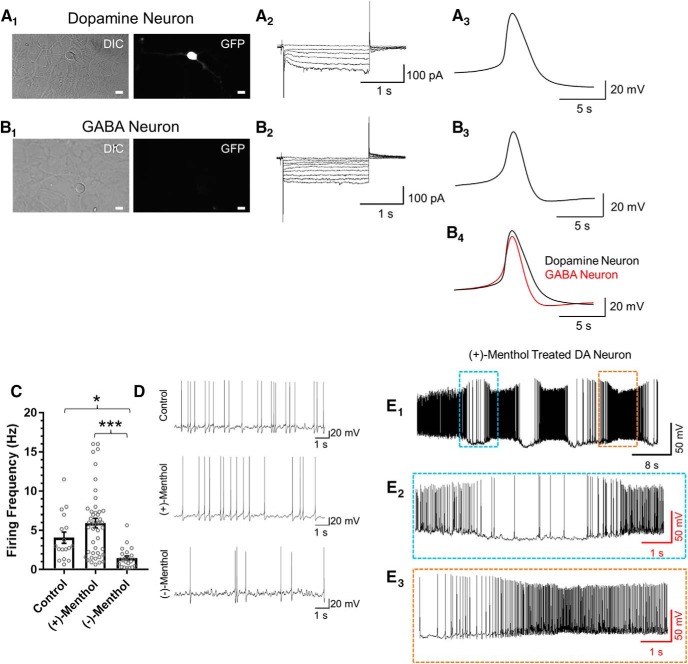
Menthol stereoisomers cause different effects on dopamine neuron baseline firing frequency. ***A_1_***, ***B_1_***, Representative image of a TH-positive cultured midbrain dopamine neuron (***A_1_***) and a TH-negative putative GABA neuron (***B_1_***). Scale bars, 20 µm. ***A_2_***, ***B_2_***, Representative waveforms of a TH-positive dopamine neuron with *I*_h_ (***A_2_***) and a TH-negative putative GABA neuron without *I*_h_ (***B_2_***). ***A_3_***, ***B_3_***, ***B_4_***, Representative action potential from cultured dopamine and GABA neurons. ***C***, Baseline firing frequency of TH-positive dopamine neurons treated with control, (+)-menthol, or (−)-menthol (500 nm, each) for 10 d. Data are the mean ± SEM. *p < 0.05; **p < 0.01 (one way ANOVA with Tukey). Circles overlaid with bars represent individual recordings that constitute the mean value for each respective group (*n* = 17, 44, and 19 for control, (+)-menthol, and (−)-menthol, respectively). Exact *p* values are provided in the text. Full data are plotted as a scatterplot with mean ± SEM values plotted as an overlaid bar chart. ***D***, Representative whole-cell current-clamp traces for TH-positive dopamine neurons treated with control or menthol stereoisomers. ***E_1_–E_3_***, 9% of the (+)-menthol-treated dopamine neurons displayed dramatic variances in firing frequency. ***E_2_*** and ***E_3_*** are magnifications of blue and orange boxes, respectively, in ***E_1_***.

**Figure 3.**
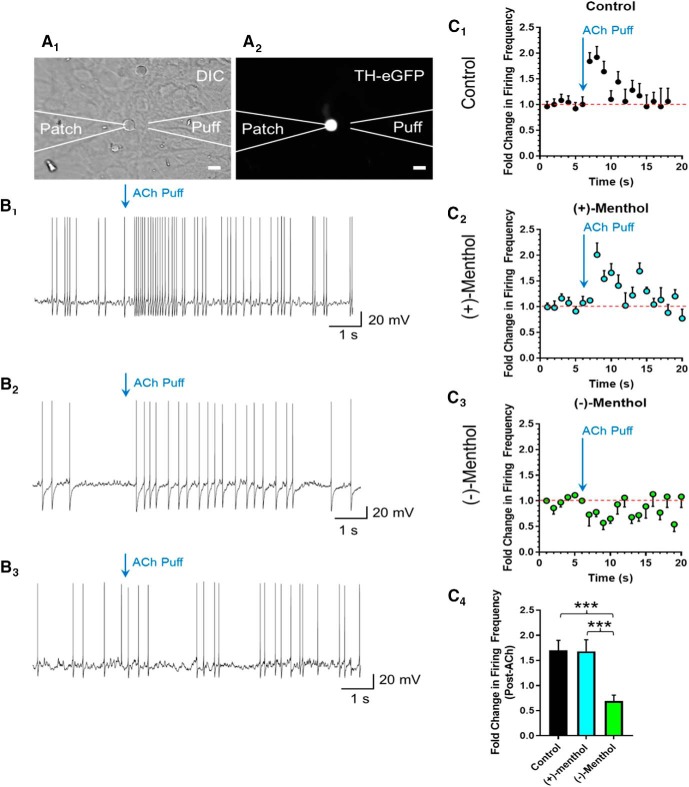
Chronic treatment with menthol stereoisomers causes different effects on dopamine neuron excitability. ***A_1_***, ***A_2_***, Representative image of TH-positive dopamine neuron with a diagram of the typical placement of patch and puffer pipets. ***B_1_–B_3_***, Representative whole-cell current-clamp recordings from TH-positive dopamine neurons treated with control, (+)-menthol, or (−)-menthol (500 nm each) for 10 d. Arrows indicate a 300 ms application of 300 µm ACh to stimulate nAChRs. ***C_1_–C_3_***, Mean firing frequency over time plot of TH-positive dopamine neurons before and after the ACh puff (indicated by arrow). ***C_4_***, Quantification of firing frequency of dopamine neurons for the 3 s after ACh puff. Data are the mean ± SEM. *n* = 5–9 TH-positive dopamine neurons.

All results are presented as the mean ± SEM, and all statistical analyses were performed using GraphPad Prism. For microscopy assays (Fig. 1) and electrophysiology using cultured neurons (Figs. 2, 3), we used a power analysis (G*Power software, www.gpower.hhu.de) to determine appropriate sample sizes. In experiments that involved three or more group comparisons (Figs. 1–3), data were analyzed using a one-way or a two-way ANOVA. The choice of a one-way or two-way ANOVA is listed with the corresponding experiment in the Results section. When effects were shown to be significant, a *post hoc* analysis (Tukey’s test) was performed to compare the individual group means.

## Results

### Long-term effects of menthol stereoisomers cause different effects on α4β2 nAChR upregulation

A previous report found that (±)-menthol alone upregulates α4* and α6* nAChRs in cultured neuro-2a cells and mouse midbrain dopamine neurons (Henderson et al., 2016). In the previous report, 500 nm (±)-menthol achieved robust upregulation of α4-SEPβ2 nAChRs, while 50 nm (±)-menthol had no effect (Henderson et al., 2016).

Similar to previous reports (Henderson et al., 2016, 2017), we used a combination of TIRFM and a pH-sensitive GFP version of α4 nAChR subunits (α4-SEP) to determine how (+)-menthol and (−)-menthol may act differently to upregulate nAChRs transiently transfected into neuro-2a cells (Fig. 1). This combination of TIRFM and SEP-labeled nAChRs has been used previously to study the upregulation of nAChRs (Srinivasan et al., 2011; Henderson et al., 2014, 2016, 2017). A concentration range of (+)-menthol and (−)-menthol (0.05–4 µm) was used to treat neuro-2a cells transiently transfected with α4-SEP and β2 nAChR subunits for 24 h before imaging sessions. The upregulation of α4β2 nAChRs was examined by quantifying changes in the plasma membrane raw integrated density (PMRID), which indicates a change in the number of receptors that reside on the PM. With all concentrations, (+)-menthol neither increased nor decreased the PMRID of α4-SEPβ2 nAChRs (Fig. 1). (−)-Menthol produced a significant increase in α4-SEPβ2 nAChR PMRID at concentrations of 0.1, 0.5, and 1 µm when compared with control (Fig. 1; *p* = 0.03, one-way ANOVA with *post hoc* Tukey’s test). The effect of (+)-menthol and (−)-menthol on α4β2 nAChR PMRID was significant by two-way ANOVA (*F*_(1,381)_ = 30.3, *p* = 0.0001). These results suggest that the previous effects we observed with (±)-menthol (Henderson et al., 2016, 2017) may be primarily mediated by (−)-menthol, and not (+)-menthol.

### Long-term treatment with menthol stereoisomers causes different effects on dopamine neuron firing frequency

We previously used whole-cell patch-clamp electrophysiology to report that long-term treatment of (±)-menthol decreases the baseline firing of midbrain dopamine neurons in both mouse brain slices and cultured mouse midbrain neurons (Henderson et al., 2016). To further characterize different effects of menthol stereoisomers, we conducted similar studies with cultured mouse midbrain dopamine neurons treated for 10 d with (+)-menthol or (−)-menthol (500 nm; Figs. 2, 3). To facilitate the identification of midbrain dopamine neurons, we cultured neurons from TH-eGFP mice (Fig. 2*A1–B3*; Srinivasan et al., 2016; Henderson et al., 2017). The presence of hyperpolarization-activated inwardly rectifying nonspecific cation current (*I*_h_) and spike duration was also assessed to aid in the identification of dopamine neurons. For all TH-eGFP-positive neurons (Fig. 2*A1–A3*), we detected the presence of *I*_h_ and spike durations >4 ms.

We examined cultured dopamine neurons for changes in baseline firing frequency following 10 d of treatment with either 500 nm (+)-menthol or 500 nm (−)-menthol (Fig. 2*C*,*D*). Control neurons exhibited a mean firing frequency of 4.0 ± 0.7 Hz (Fig. 2*C*). Long-term treatment with (−)-menthol produced a significant decrease in dopamine neuron mean firing frequency to 1.5 ± 0.3 Hz (*p* = 0.04, one-way ANOVA with Tukey’s test). This change in firing frequency is similar to the effect we reported following long-term treatment with (±)-menthol (Henderson et al., 2016). Long-term treatment with (+)-menthol did not produce a significant change in dopamine neuron firing frequency (Fig. 2*C*,*D*). We did observe that 20% of neurons treated with (+)-menthol exhibited an increase in firing frequency. All neurons exhibiting an increase in firing frequency were positive for TH-eGFP fluorescence, *I*_h_, and spike duration >4 ms. Therefore, we are confident that these neurons are indeed dopamine neurons and not GABA neurons, which are also present in these cultured midbrain neuron preparations. Further examination showed that a few of these neurons (10% of the 20%) exhibited spontaneous variations in baseline firing frequency (Fig. 2*E1–E3*).

### Long-term treatment with menthol stereoisomers causes different effects on dopamine neuron excitability

We previously reported that (±)-menthol alters dopamine neuron excitability (Henderson et al., 2016). Dopamine neurons exhibit increases in firing frequency when exposed to a nAChR agonist (Nashmi et al., 2007; Drenan et al., 2008). When menthol is combined with nicotine, it can potentiate this effect (Henderson et al., 2017). Despite this, long-term treatment with menthol alone decreased dopamine neuron excitability (Henderson et al., 2016). Using similar methods, we investigated both menthol stereoisomers for their ability to alter dopamine neuron excitability. Similar to the previous experiments, we used TH-eGFP midbrain cultures and treated them for 10 d with (+)-menthol or (−)-menthol (both at 500 nm). We recorded dopamine neurons in current-clamp mode and applied a 300 ms puff of 300 µm ACh to stimulate nAChRs (Fig. 3). A concentration of 300 µm ACh was selected to stimulate a peak response from the nAChRs residing on DA neurons. Control dopamine neurons exhibited a transient, twofold ACh-induced increase in firing frequency that was similar to previous reports (Henderson et al., 2016; Fig. 3*B1*,*C1*). Dopamine neurons exposed to (+)-menthol exhibited a similar 1.9-fold transient ACh-induced increase in firing frequency (Fig. 3*B2*,*C2*). In contrast, dopamine neurons treated with (−)-menthol exhibited a consistent ACh-induced decrease in firing frequency (Fig. 3*B3*,*C3*,*C4*). When we compared the fold change in firing frequency following ACh application, we observed a significant effect of treatment with menthol stereoisomers (one-way ANOVA, *F*_(2,17)_ = 9.96, *p* = 0.0014). The change in dopamine neuron firing frequency following long-term treatment with (−)-menthol was significant when compared with both control-treated and (+)-menthol-treated dopamine neurons (*p* = 0.002 and *p* = 0.007, respectively). These data further support the suggestion that (−)-menthol is the primary mediator for the effects we previously observed with (±)-menthol.

### Acute applications of menthol stereoisomers exhibit similar pharmacologic profiles

**Figure 4.**
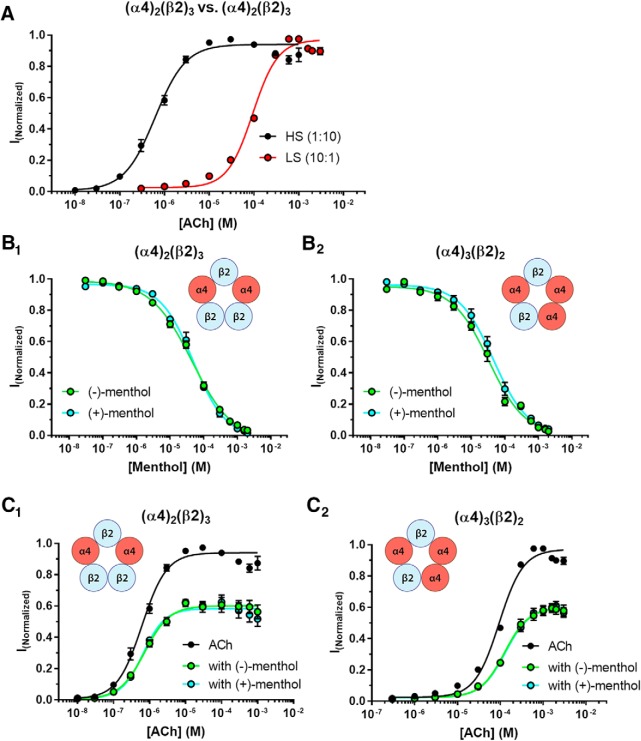
Acute applications display only slight pharmacological differences among menthol stereoisomers. ***A***, Concentration–response curves of ACh on oocytes injected with α4 and β2 nAChR subunits. Injections were biased to assemble high-sensitivity (α4)_2_(β2)_3_ nAChRs (1:10) or low-sensitivity (α4)_3_(β2)_2)_ nAChRs (10:1). ***B_1_***, ***B_2_***, Concentration–response curve of menthol stereoisomers with high sensitivity (α4)_2_(β2)_3_ nAChRs (***B_1_***) or low sensitivity (α4)_3_(β2)_2_ nAChRs (***B_2_***). ***C_1_***, ***C_2_***, Concentration–response curve of ACh in the absence or presence of (−)-menthol or (+)-menthol (50 µm) with high sensitivity (α4)_2_(β2)_3_ nAChRs (***C_1_***) or low sensitivity (α4)_3_(β2)_2_ nAChRs (***C_2_***). Refer to Tables 1–3 for values for Hill coefficient, EC_50_, and IC_50_.

Our long-term applications of menthol have studied submicromolar concentrations that we perceive to be pharmacologically relevant (Henderson et al., 2016). Pioneering observations showed that menthol at higher concentrations (100- to 1000-fold greater than the concentration we use in our long-term studies) acts as a negative allosteric modulator of α4β2 nAChRs (Hans et al., 2012), a noncompetitive antagonist of α3β4 nAChRs (Ton et al., 2015), and a noncompetitive antagonist of α7 nAChRs (Ashoor et al., 2013b). Although the concentrations used in these studies may not be pharmacologically relevant in some cases, this profile suggests that menthol directly interacts with nAChRs and most likely at an allosteric site. Using *X. laevis* oocytes and two-electrode voltage-clamp electrophysiology, we used concentration–response assays to examine how acute applications of (+)-menthol and (−)-menthol differed in their ability to act as inhibitors of α4β2 nAChR function (Fig. 4).

Long-term treatment with nAChR ligands can change the stoichiometry of α4β2 nAChRs. Thus, we studied menthol inhibition on both the low-sensitivity and high-sensitivity stoichiometries of α4β2 nAChRs [(α4)_3_(β2)_2_ and (α4)_2_(β2)_3_, respectively; Fig. 4]. On both α4β2 nAChR stoichiometries, we observed potencies and efficacies for ACh that are similar to previously reported data (Fig. 4*A*, Table 1; Nelson et al., 2003; Tapia et al., 2007). Both (+)-menthol and (−)-menthol inhibited both stoichiometries of α4β2 nAChRs (Fig. 4*B1*,*B2*, Table 2). (−)-Menthol was modestly more potent at both stoichiometries (*p* = 0.033 and *p* = 0.0001 for (α4)_2_(β2)_3_ and (α4)_3_(β2)_2_ nAChRs, respectively; Table 2). We also compared (+)-menthol and (−)-menthol with regard to competitive versus noncompetitive actions. Using methods similar to those of Hans et al. (2012), we used a concentration response of ACh in the absence and presence of menthol stereoisomers (50 µm) to test whether menthol caused insurmountable inhibition (a type of noncompetitive inhibition) or a parallel shift to the right in EC_50_ (indicative of competitive inhibition; Fig. 4*C1*,*C2*). Here both (+)-menthol and (−)-menthol caused a 40% decrease in the efficacy of ACh with no significant change in EC_50_ (Table 3, Fig. 4*C1*,*C2*). From this, both menthol stereoisomers act as noncompetitive inhibitors of α4β2 nAChRs with similar potency despite the clear difference we observed with their long-term, low-dose applications.

**Table 1:** Concentration–response relationships of ACh on (α4)_3_(β2)_2_ and (α4)_2_(β2)_3_ nAChRs

	n_H_	EC_50_ (μm)	*n*	I_max_ (μA)
1:10 (α4)_2_(β2)_3_	1.54 ± 0.08	93.4 ± 3.34	10	0.3 – 1.1
10:1 (α4)_3_(β2)_2_	1.27 ± 0.10	0.62 ± 0.04	15	0.2 – 13.6

**Table 2:** : Concentration–response relationships of menthol stereoisomers on (α4)_3_(β2)_2_ and (α4)_2_(β2)_3_ nAChRs

	n_H_	IC_50_ (μm)	*n*	I_max_ (μA)
1:10 (α4)_2_(β2)_3_ ^*a*^
(–)-Menthol	−0.71 ± 0.02	44.9 ± 1.62	10	0.08–1.34
(+)-Menthol	−0.90 ± 0.03	48.3 ± 1.67^*c*^	10	0.12–0.97
10:1 (α4)_3_(β2)_2_ ^*b*^
(–)-Menthol	−0.81 ± 0.03	33.1 ± 1.83	10	0.09–1.30
(+)-Menthol	−0.85 ± 0.03	45.5 ± 2.03^*d*^	10	0.13–1.37

a Stimulated with 2 µm ACh.

b Stimulated with 100 µm ACh.

c *p* = 0.033, IC_50_ of (−)-menthol vs. (+)-menthol on (α4)_2_(β2)_3_ nAChRs.

d *p* = 0.0001, IC_50_ of (−)-menthol vs. (+)-menthol on (α4)_2_(β2)_3_ nAChRs.

**Table 3:** Concentration–response of ACh in the absence and presence of menthol stereoisomers on (α4)_3_(β2)_2_ and (α4)_2_(β2)_3_ nAChRs

Receptor	n_H_	IC_50_ (μm)	*n*	I_max_ (μA)
1:10 (α4)_2_(β2)_3_ ^*a*^
ACh only	1.27 ± 0.05	0.61 ± 0.0205	15	0.04–1.05
With (–)-menthol	1.38 ± 0.08	0.66 ± 0.0318	12	0.05–0.82
With (+)-menthol	1.51 ± 0.10	0.64 ± 0.0321	15	0.03–0.85
10:1 (α4)_3_(β2)_2_ ^*b*^
ACh only	1.74 ± 0.07	99.8 ± 2.37	19	0.2–13.6
With (–)-menthol	1.58 ± 0.04	120.8 ± 2.37	18	0.1–9.9
With (+)-menthol	1.56 ± 0.04	126.4 ± 2.52	19	0.1–9.3

a Stimulated with 2 µm ACh.

b Stimulated with 100 µm ACh.

### Flooding simulations suggest a putative menthol binding site on α4β2 nAChRs

**Figure 5.**
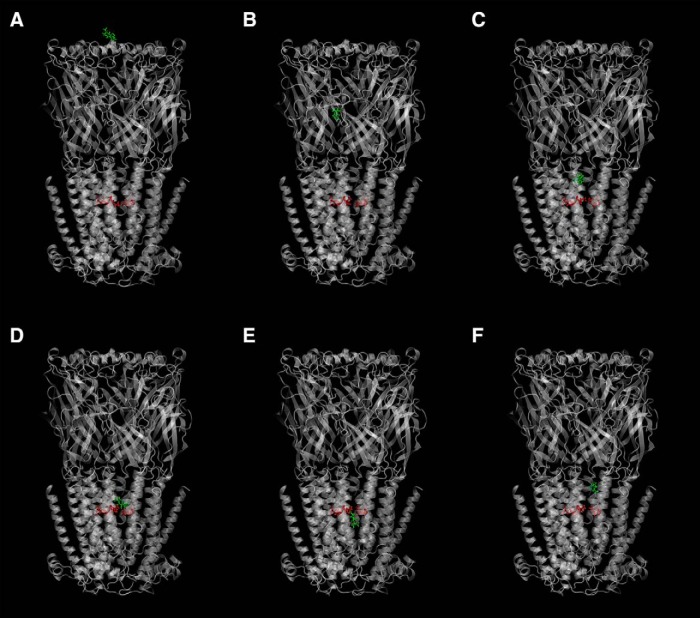
Snapshots of a menthol molecule during flooding simulation. ***A***, Menthol (shown in green sticks) is introduced in the extracellular solution. Menthol first encounters the extracellular domain (ECD) of the human α4β2 nicotinic receptor. ***B***, Menthol binds to different residues on the ECD. ***C***, Menthol reaches the “top” of the M2 region. ***D–F***, For the rest of the flooding simulation, menthol remains in the transmembrane domain, between the “top” of the M2 region and the ring of 9´ Leu residues (shown as red sticks). Menthol does not move toward the cytoplasmic end of M2. The full movie may be downloaded from https://drive.google.com/drive/folders/1R7PWDpd7QpffZt7r9b3BWlH73WZgNbH0?usp=sharing.

**Figure 6.**
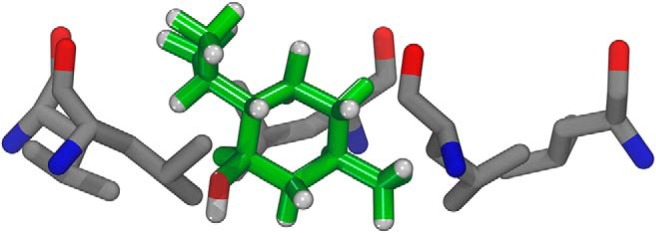
A snapshot of a menthol molecule sitting in the center of the ring of 9´ leucine residues. The carbons of menthol are shown in green, its hydrogens in white, and its oxygen in red. The carbons of the 9´ leucine residues are shown in gray, their oxygens in red, and their nitrogens in blue. The hydrogens of the leucine residue are not shown in the figure. The image is a higher-resolution snapshot from the movie available at https://drive.google.com/file/d/1eETqhvRk50-UayXNfUG94sHSwxSK6OoC/view.

As computational resources become more efficient, more studies are using molecular dynamics to guide experimental design (Ashoor et al., 2013b; Dalton et al., 2014; Arcario et al., 2017; Bottaro and Lindorff-Larsen, 2018). We used “flooding” simulations in which copies of menthol were added to the solution phase of a simulation system containing an α4β2 nAChR (Brannigan et al., 2010). Most of the menthol molecules partition to the lipid membrane, leaving just a small percentage of the total number of menthol molecules in the aqueous phase. The protein remained stable throughout the simulation and predicted binding sites were deemed notable if menthol remained within 5 Å of the protein for >50 ns. Many of these predicted binding sites were on the periphery of the protein; however, one was inside the receptor pore (Fig. 5). Specifically, the site in the pore was the ring of leucines at the 9´ position on TM2 (Fig. 6). This position is important for modulating the sensitivity of the protein to agonists without affecting the agonist-binding site (Labarca et al., 1995; Kearney et al., 1996; Kosolapov et al., 2000). Based on the simulation and the importance of the 9´ site, we probed this prediction further with oocyte expression experiments (Tables 4, 5). We also probed many of the other predicted binding sites on the periphery of the protein, but our results showed that mutating these sites had no meaningful influence on menthol potency (Tables 6, 7). Among these mutants, we observed no detectable currents from (α4[E182A])_3_(β2[wt])_2_, (α4[D51A, E52A])_3_(β2[wt])_2_, (α4[Q55A])_3_(β2[wt])_2_, (α4[I280A])_3_(β2[wt])_2_, (α4[I226A])_3_(β2[wt])_2_, (α4[L279A])_3_(β2[wt])_2_, (α4[Y283A])_3_(β2[wt])_2_, (α4[L229A])_3_(β2[wt])_2_, (α4[G281W])_3_(β2[wt])_2_, (α4[E282D])_3_ (β2[wt])_2_, (α4[E282A])_3_(β2[wt])_2_, and (α4[wt])_3_ (β2[I214A])_2_ when 1 mm ACh was applied. Additionally, IC_50_ data were not trustworthy for (−)-menthol for (α4[wt])_3_ (β2[G364L])_2_, (α4[wt])_3_ (β2[F306W])_2_, (α4[M288W])_3_ (β2[wt])_2_, (α4[wt])_3_ (β2[M280W])_2_, and (α4[V356W])_3_ (β2[M368W])_2_ because currents were too small for systematic measurements at approximately half-maximal [ACh]. We did not calculate IC_50_ values for (−)-menthol for (α4[L9´A])_3_ (β2[M368W])_2_, (α4[L9´T])_3_ (β2[wt])_2_, (α4[L9´A])_3_ (β2[wt])_2_, α4[L9´A])_3_ (β2[V360W])_2_, and (α4[L9´A])_3_ (β2[F328W])_2_ because inhibition with (−)-menthol was too weak for systematic fitting to the Hill equation. We probed only a subset of residues with (+)-menthol for stereoselectivity comparison experiments.

**Table 4:** **: IC_40_ values for** (−)**-menthol on (α4[L**9´**X])_2_(β2)_3_ mutant nAChRs**

Residue	IC_30_ (µm (+)-menthol ± SEM)	*n*
Ala	855 ± 130	18
Ser	357 ± 62.0	11
Gln	117 ± 14.0	13
Thr	547 ± 110	11
Cys	162 ± 23.0	11
Ile	50.1 ± 11.0	7
Met	18.4 ± 1.60	10
Leu (WT)	35.6 ± 5.70	9
Phe	121 ± 10.0	10
Trp	26.6 ± 4.90	10

**Table 5:** **: IC_30_ values for (+)-menthol on (α4[L**9´**X])_2_(β2)_3_ mutant nAChRs**

Residue	IC_30_ (µm (+)-menthol ± SEM)	*n*
Ala	760 ± 100	9
Ser	215 ± 41.0	11
Gln	114 ± 12.0	10
Thr	475 ± 47.0	9
Cys	108 ± 28.0	11
Ile	70.4 ± 18.0	9
Met	15.6 ± 5.50	15
Leu (WT)	27.1 ± 4.50	8
Phe	60.0 ± 12.0	8
Trp	11.3 ± 1.60	18

**Table 6:** EC_50_ values for mutant α4β2 nAChRs

Motivation	Mutant	EC_50_ (µm ACh) ± SEM	Hill coefficient ± SEM
WT-LS	(α4[wt])_3_(β2[wt])_2_	83.4 ± 4.67	0.905 ± 0.03
WT-HS	(α4[wt])_2_(β2[wt])_3_	0.924 ± 0.0656	0.924 ± 0.1
WT-α5	α5[wt](α4[wt]β2[wt])_2_	0.409 ± 0.0418	1.06 ± 0.1
9´ residue	(α4[L9´A])_2_(β2[wt])_3_	0.499 ± 0.032	1.43 ± 0.1
9´ residue	(α4[L9´S])_2_(β2[wt])_3_	0.0356 ± 0.00145	1.29 ± 0.06
9´ residue	(α4[L9´I])_2_(β2[wt])_3_	960. ± 502	0.489 ± 0.03
9´ residue	(α4[L9´T])_2_(β2[wt])_3_	0.282 ± 0.0233	1.28 ± 0.1
9´ residue	(α4[L9´Q])_2_(β2[wt])_3_	0.530 ± 0.0136	1.284 ± 0.03
9´ residue	(α4[L9´C])_2_(β2[wt])_3_	2.68 ± 0.311	0.710 ± 0.1
9´ residue	α4[L9´F])_2_(β2[wt])_3_	0.547 ± 0.0209	1.26 ± 0.05
9´ residue	(α4[L9´W])_2_(β2[wt])_3_	0.240 ± 0.00906	1.19 ± 0.05
GABA	(α4[L376W])_3_(β2[wt])_2_	247 ± 99.4	0.772 ± 0.1
MD	(α4[V356A])_3_(β2[wt])_2_	244 ± 113	0.843 ± 0.2
GABA	(α4[wt])_3_(β2[M305W])_2_	180. ± 38.2	0.938 ± 0.1
MD	(α4[wt])_3_(β2[W355L])_2_	171 ± 28.0	1.04 ± 0.1
MD	(α4[W363L])_3_(β2[wt])_2_	159 ± 55.2	0.941 ± 0.2
GABA	(α4[wt])_3_(β2[G364L])_2_	153 ± 164	0.728 ± 0.3
TRPM8	(α4[L383A])_3_(β2[wt])_2_	150. ± 7.67	0.919 ± 0.03
TRPM8	(α4[wt])_3_(β2[R299A])_2_	140. ± 64.7	0.510 ± 0.05
MD	(α4[I357A])_3_(β2[wt])_2_	124 ± 19.1	0.957 ± 0.09
MD	(α4[W363L])_3_(β2[W355L])_2_	122 ± 11.6	1.31 ± 0.1
GABA	(α4[wt])_3_(β2[F306W])_2_	106 ± 12.2	1.07 ± 0.1
ADNFLE	(α4[S6'F])_3_(β2[wt])_2_	105 ± 47.5	1.27 ± 0.1
MD	(α4[F299A])_3_(β2[wt])_2_	83.2 ± 7.35	1.22 ± 0.1
9´ residue	(α4[L9´O-Me-Thr])_3_(β2[wt])_2_	61.3 ± 4.64	1.07 ± 0.07
GABA	(α4[wt])_3_(β2[L282W])_2_	77.5 ± 6.44	1.01 ± 0.06
GABA	(α4[wt])_3_(β2[V360W])_2_	71.2 ± 5.79	1.24 ± 0.1
GABA	(α4[M288W])_3_(β2[wt])_2_	70.8 ± 11.1	0.913 ± 0.1
GABA	(α4[wt])_3_(β2[M280W])_2_	57.8 ± 6.94	1.29 ± 0.2
ADNFLE	(α4[wt])_3_(β2[V287L])_2_	38.0 ± 23.8	0.493 ± 0.07
Docking	(α4[G281A, V236A])_3_(β2[wt])_2_	28.1 ± 4.84	0.998 ± 0.1
Docking	(α4[wt])_3_(β2[I218A,L257A])_2_	26.6 ± 3.65	0.862 ± 0.08
Docking + GABA	(α4[G281A, V236A])_3_(β2[M368W])_2_	24.3 ± 5.55	0.629 ± 0.07
GABA	(α4[wt])_3_(β2[Y275W])_2_	21.1 ± 6.75	0.988 ± 0.1
GABA	(α4[wt])_3_(β2[M368W])_2_	1.58 ± 0.508	0.493 ± 0.09
MD + GABA	(α4[V356W])_3_(β2[M368W])_2_	1.46 ± 0.219	1.56 ± 0.3
# 9´A	α5[V9´A](α4[wt]β2[wt])_2_	0.818 ± 0.0178	0.818 ± 0.03
ADNFLE	(α4[S10'L])_3_(β2[wt])_2_	0.671 ± 0.112	0.512 ± 0.04
MD + GABA	(α4[F299W, L376W])_3_(β2[M368W])_2_	0.511 ± 0.0293	1.39 ± 0.09
MD	(α4[F299W])_3_(β2[wt])_2_	0.508 ± 0.0309	1.20 ± 0.07
MD + GABA	(α4[L376W])_3_(β2[M368W])_2_	0.504 ± 0.0711	1.41 ± 0.2
9´ residue	(α4[L9´M])_3_(β2[wt])_2_	0.503 ± 0.0663	0.585 ± 0.04
MD + GABA	(α4[F299W])_3_(β2[M368W])_2_	0.486 ± 0.0434	1.25 ± 0.1
9´ residue	(α4[L9´M])_2_(β2[wt])_3_	0.380 ± 0.0307	1.22 ± 0.1
MD	(α4[V356W])_3_(β2[wt])_2_	0.360 ± 0.0188	1.65 ± 0.1
9´ residue + GABA	(α4[L9´A])_3_(β2[M368W])_2_	0.0230 ± 0.00200	0.900 ± 0.06
# 9´A	(α4[wt])_3_(β2[L9´A])_2_	0.0695 ± 0.00353	1.32 ± 0.07
9´ residue	(α4[L9´T])_3_(β2[wt])_2_	0.0981 ± 0.00729	0.995 ± 0.06
9´ residue	(α4[L9´I])_3_(β2[wt])_2_	160. ± 50.3	0.866 ± 0.1
9´ residue	(α4[wt])_2_(β2[L9´A])_3_	0.0197 ± 0.00163	1.12 ± 0.09
9´ residue	(α4[L9´A])_3_(β2[wt])_2_	0.0159 ± 0.000691	1.16 ± 0.05
9´ residue + MD	(α4[L9´A])_3_(β2[V360W])_2_	0.00352 ± 0.000290	1.19 ± 0.1
9´ residue + MD	(α4[L9´A])_3_(β2[F328W])_2_	0.00318 ± 0.000237	1.13 ± 0.08
ACh Binding Site	(α4[H116V,Q124F,T126L])_3_(β2[wt])_2_	76.3 ± 1.85	1.63 ± 0.06
Intra-subunit	(α4[C233A])_3_(β2[wt])_2_	143 ± 17.2	0.99 ± 0.08
Intra-subunit	(α4[T235A])_3_(β2[wt])_2_	43.6 ± 3.89	1.10 ± 0.09
Intra-subunit	(α4[V236A])_3_(β2[wt])_2_	76.2 ± 14.2	1.31 ± 0.3
Intra-subunit	(α4[G281F])_3_(β2[wt])_2_	33.5 ± 9.87	1.49 ± 0.6
Intra-subunit	(α4[S232A])_3_(β2[wt])_2_	162 ± 20.6	0.993 ± 0.08
Intra-subunit	(α4[G281A])_3_(β2[wt])_2_	19.9 ± 1.47	1.49 ± 0.2
Near Cys loop	(α4[E52L])_3_(β2[wt])_2_	33.3 ± 2.71	1.27 ± 0.1
Near Cys loop	(α4[E182Q])_3_(β2[wt])_2_	63.5 ± 4.54	1.66 ± 0.2
Near Cys loop	(α4[wt])_3_(β2[S44A])_2_	86.1 ± 8.21	1.70 ± 0.3
Channel pore	(α4[L265A])_3_(β2[wt])_2_	26.5 ± 6.58	0.864 ± 0.2
Channel pore	(α4[wt])_3_(β2[L257A])_2_	0.316 ± 0.101	1.31 ± 0.4
Interfacial	(α4[F286A])_3_(β2[wt])_2_	46.5 ± 8.45	0.962 ± 0.1
Interfacial	(α4[wt])_3_(β2[I218A])_2_	119 ± 11.7	1.21 ± 0.1
Interfacial	(α4[G281A])_3_(β2[I218A])_2_	23.8 ± 2.28	0.868 ± 0.5

WT-LS, Wild-type α4β2 receptor with the low sensitivity stoichiometry; WT-HS, wild-type α4β2 receptor with the high sensitivity stoichiometry; WT-α5, wild-type α4β2 receptor with α5 in the auxiliary position; 9´ residue, a mutant at the 9´ position to probe the effects of mutations at this site; GABA, analogous site for the menthol binding site in the GABA receptor; MD, molecular dynamics predicted position; TRPM8, analogous site for the menthol binding site on the TRPM8 receptor; ANDFLE, a mutant that is found in ADNFLE patients; Docking, position predicted by preliminary docking studies; #L9´A, mutant made to probe the effects that the number of 9´A mutations has on the effects of menthol; ACh binding site, the putative ACh binding site; Intra-subunit, site between subunits in the transmembrane domain of α4β2; Near Cys loop, site that is near the Cys loop of α4β2; Channel pore, site that is in the channel pore of α4β2; Interfacial, a site on the transmembrane domain of a single subunit that faces toward the other transmembrane domain helices on the same subunit.

**Table 7:** IC_50_ values for mutant α4β2 nAChRs

Motivation	Mutant	IC_50_ (µm (−)-menthol) ± SEM	Hill coefficient ± SEM	Maximum inhibition (%)
WT-LS	(α4[wt])_3_(β2[wt])_2_	33.7 ± 1.16	0.952 ± 0.04	95
WT-HS	(α4[wt])_2_(β2[wt])_3_	33.9 ± 7.40	0.481 ± 0.1	71
WT-α5	α5[wt](α4[wt]β2[wt])_2_	51.0 ± 9.06	0.593 ± 0.2	53
9´ residue	(α4[L9´A])_2_(β2[wt])_3_	114 ± 152	0.779 ± 0.2	45
9´ residue	(α4[L9´S])_2_(β2[wt])_3_	514 ± 361	0.740 ± 0.1	65
9´ residue	(α4[L9´I])_2_(β2[wt])_3_	97.3 ± 38.5	0.836 ± 0.2	79
9´ residue	(α4[L9´T])_2_(β2[wt])_3_	717 ± 770.	0.946 ± 0.3	59
9´ residue	(α4[L9´Q])_2_(β2[wt])_3_	165 ± 28.1	1.03 ± 0.1	81
9´ residue	(α4[L9´C])_2_(β2[wt])_3_	306 ± 173	0.744 ± 0.1	65
9´ residue	α4[L9´F])_2_(β2[wt])_3_	211 ± 75.6	1.01 ± 0.1	72
9´ residue	(α4[L9´W])_2_(β2[wt])_3_	28.8 ± 3.26	0.917 ± 0.1	87
GABA	(α4[L376W])_3_(β2[wt])_2_	17.4 ± 43.2	2.38 ± 1.6	92
MD	(α4[V356A])_3_(β2[wt])_2_	29.6 ± 11.0	0.631 ± 0.2	80
GABA	(α4[wt])_3_(β2[M305W])_2_	70.6 ± 29.9	0.872 ± 0.2	90
MD	(α4[wt])_3_(β2[W355L])_2_	28.8 ± 24.6	0.589 ± 0.4	80
MD	(α4[W363L])_3_(β2[wt])_2_	42.7 ± 9.37	1.02 ± 0.2	96
TRPM8	(α4[L383A])_3_(β2[wt])_2_	27.9 ± 4.97	0.828 ± 0.1	96
TRPM8	(α4[wt])_3_(β2[R299A])_2_	52.0 ± 5.89	1.30 ± 0.2	96
MD	(α4[I357A])_3_(β2[wt])_2_	31.4 ± 6.23	1.30 ± 0.3	94
MD	(α4[W363L])_3_(β2[W355L])_2_	28.4 ± 6.96	0.866 ± 0.2	91
ADNFLE	(α4[S6'F])_3_(β2[wt])_2_	32.3 ± 6.05	1.19 ± 0.3	95
MD	(α4[F299A])_3_(β2[wt])_2_	20.4 ± 10.4	0.548 ± 0.2	93
9´ residue	(α4[L9´O-Me-Thr])_3_(β2[wt])_2_	135.9 ± 20.9	0.883 ± 0.7	79
GABA	(α4[wt])_3_(β2[L282W])_2_	26.1 ± 5.40	0.883 ± 0.1	97
GABA	(α4[wt])_3_(β2[V360W])_2_	16.7 ± 1.37	1.08 ± 0.09	96
ADNFLE	(α4[wt])_3_(β2[V287L])_2_	17.0 ± 5.67	0.766 ± 0.2	89
Docking	(α4[G281A, V236A])_3_(β2[wt])_2_	11.0 ± 0.767	0.958 ± 0.06	96
Docking	(α4[wt])_3_(β2[I218A,L257A])_2_	23.0 ± 2.68	1.57 ± 0.3	99
Docking + GABA	(α4[G281A, V236A])_3_(β2[M368W])_2_	59.5 ± 15.2	0.891 ± 0.2	95
GABA	(α4[wt])_3_(β2[Y275W])_2_	21.7 ± 5.21	1.06 ± 0.2	97
GABA	(α4[wt])_3_(β2[M368W])_2_	57.8 ± 8.07	0.865 ± 0.09	93
# 9´A	α5[V9´A](α4[wt]β2[wt])_2_	1750 ± 3360	0.907 ± 0.3	36
ADNFLE	(α4[S10'L])_3_(β2[wt])_2_	29.4 ± 4.32	1.39 ± 0.2	97
MD + GABA	(α4[F299W, L376W])_3_(β2[M368W])_2_	107 ± 7.07	1.07 ± 0.06	94
MD	(α4[F299W])_3_(β2[wt])_2_	32.5 ± 9.35	0.604 ± 0.09	87
MD + GABA	(α4[L376W])_3_(β2[M368W])_2_	107 ± 29.6	0.890 ± 0.2	87
9´ residue	(α4[L9´M])_3_(β2[wt])_2_	39.2 ± 7.98	0.848 ± 0.09	83
MD + GABA	(α4[F299W])_3_(β2[M368W])_2_	92.5 ± 36.8	0.777 ± 0.2	79
9´ residue	(α4[L9´M])_2_(β2[wt])_3_	26.6 ± 1.90	1.04 ± 0.07	91
MD	(α4[V356W])_3_(β2[wt])_2_	52.6 ± 43.6	0.599 ± 0.2	70
# 9´A	(α4[wt])_3_(β2[L9´A])_2_	1920 ± 9620	0.807 ± 0.6	38
9´ residue	(α4[L9´I])_3_(β2[wt])_2_	127 ± 29.1	0.947 ± 0.1	84
9´ residue	(α4[wt])_2_(β2[L9´A])_3_	3030 ± 2480	0.832 ± 0.8	31
ACh binding site	(α4[H116V,Q124F,T126L])_3_(β2[wt])_2_	64.4 ± 2.09	1.20 ± 0.05	90
Intra-subunit	(α4[C233A])_3_(β2[wt])_2_	46.1 ± 6.43	1.53 ± 0.3	98
Intra-subunit	(α4[T235A])_3_(β2[wt])_2_	66.6 ± 8.03	1.60 ± 0.3	97
Intra-subunit	(α4[V236A])_3_(β2[wt])_2_	58.9 ± 10.0	1.79 ± 0.4	91
Intra-subunit	(α4[G281F])_3_(β2[wt])_2_	108 ± 19.1	1.14 ± 0.2	99
Intra-subunit	(α4[S232A])_3_(β2[wt])_2_	92.0 ± 26.7	1.64 ± 0.7	92
Intra-subunit	(α4[G281A])_3_(β2[wt])_2_	25.6 ± 1.43	1.49 ± 0.1	99
Near Cys Loop	(α4[E52L])_3_(β2[wt])_2_	22.7 ± 1.67	0.969 ± 0.07	96
Near Cys loop	(α4[E182Q])_3_(β2[wt])_2_	24.3 ± 0.593	1.23 ± 0.04	81
Near Cys loop	(α4[wt])_3_(β2[S44A])_2_	59.9 ± 5.09	2.74 ± 0.6	94
Channel pore	(α4[L265A])_3_(β2[wt])_2_	21.1 ± 3.06	1.16 ± 0.2	97
Channel pore	(α4[wt])_3_(β2[L257A])_2_	74.8 ± 15.6	1.36 ± 0.3	98
Interfacial	(α4[F286A])_3_(β2[wt])_2_	54.2 ± 19.9	0.927 ± 0.3	87
Interfacial	(α4[wt])_3_(β2[I218A])_2_	60.9 ± 7.63	1.12 ± 0.1	99
Interfacial	(α4[G281A])_3_(β2[I218A])_2_	23.8 ± 1.58	2.25 ± 0.3	99

WT-LS, Wild-type α4β2 receptor with the low sensitivity stoichiometry; WT-HS, wild-type α4β2 receptor with the high sensitivity stoichiometry; WT-α5, wild-type α4β2 receptor with α5 in the auxiliary position; 9´ residue, a mutant at the 9´ position to probe the effects of mutations at this site; GABA, analogous site for the menthol binding site in the GABA receptor; MD, molecular dynamics predicted position; TRPM8, analogous site for the menthol binding site on the TRPM8 receptor; ANDFLE, a mutant that is found in ADNFLE patients; Docking, position predicted by preliminary docking studies; #L9´A, mutant made to probe the effects that the number of 9´A mutations has on the effects of menthol; ACh Binding Site, the putative ACh binding site; Intra-subunit, site between subunits in the transmembrane domain of α4β2; Near Cys Loop, site that is near the Cys loop of α4β2; Channel Pore, site that is in the channel pore of α4β2; Interfacial, a site on the transmembrane domain of a single subunit that faces toward the other transmembrane domain helices on the same subunit.

### The L9´ residue on M2 is important for menthol inhibition

**Figure 7.**
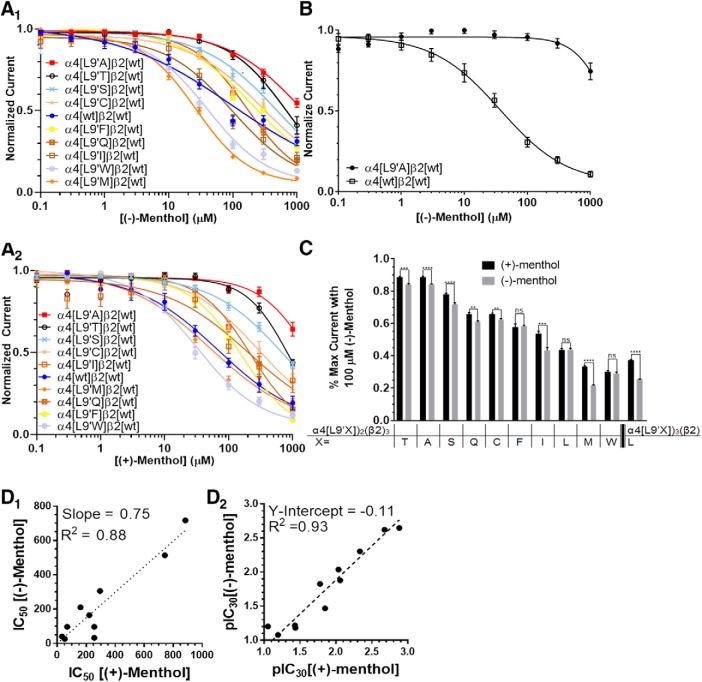
α4L9´ mutations probe the putative binding site of menthol. ***A_1_***, ***A_2_***, Concentration–response curves for menthol against (α4[L9´X])_2_(β2)_3_, where X is any amino acid. Each receptor is activated by its respective EC_50_ dose of ACh (A_1_) (−)-menthol and (A_2_) (+)-menthol. ***B***, Concentration–response curves for (α4[L9´X])_3_(β2)_2_ and (α4)_3_(β2)_2_ using 1 µm ACh. ***C***, Comparing the percentage maximum current induced when the oocyte is exposed to 100 µm menthol. *p < 0.05; **p < 0.01; ***p < 0.005; ****p < 0.001. Exact *p* values are provided in extended data (Fig. 7-1). ***D_1_***, Comparing the IC_50_ values. In this plot, (α4[L9´A])_2_(β2)_3_ is omitted because at no tested concentration of menthol was the receptor inhibited 50%. ***D_2_***, Plot of IC_30_ values comparing (+)-menthol and (−)-menthol; *n* = 6–18 oocytes.

Based on the simulation results, we made additional mutations to (α4)_2_(β2)_3_ nAChRs at the L9´ site and measured dose–response relations for (−)-menthol using two-electrode voltage-clamp electrophysiology (Fig. 7). The data confirmed that menthol block depends on the residue at the 9´ position on the M2 helix in nAChR subunits (Fig. 7). Further, mutating this position to different amino acids elicited a range of potency for menthol inhibition. The greatest effect on the ability of menthol to inhibit α4β2 nAChRs was observed with the leucine to alanine (L9´A) mutation (Fig. 7*A1*,*A2*, Tables 4, 5). The α4[L9´A]β2 receptor is ∼100-fold less sensitive to menthol block than wild type (WT; Fig. 7*B*). At the other extreme of sensitivity, α4[L9´M]β2 is more sensitive (∼10-fold). Based on these results, menthol-induced inhibition depends strongly on the side chain at the 9´ position.

10.1523/ENEURO.0465-18.2018.supplement Supplementary figure 7-1 p-values for (+)-menthol and (−)-menthol comparison between α4[L9‵X]β2[wt] mutants. Download Figure 7-1, DOCX file

A possible complication is that we tested menthol using much lower ACh for measurements on the (α4[L9´A])_3_(β2)_2_ receptor than on the WT receptor. This choice was driven by our desire to test menthol effects at roughly equally effective [ACh] for each individual receptor combination; the (α4[L9´A])_3_(β2)_2_ receptor has 10^4^-fold lower EC_50_ values for ACh when compared with the wild-type receptor (Labarca et al., 1995; Kearney et al., 1996; Kosolapov et al., 2000; Dash et al., 2014). Nonetheless, one asks whether one can treat the menthol block as truly independent of events of the agonist binding site. To address this issue, we performed additional experiments using 1 µm ACh to activate the WT and mutant receptors (Fig. 7*B*). The partially activated WT receptor is blocked ∼50% by ∼30 µm menthol; but the fully activated (α4[L9´A])_3_(β2)_2_ receptor remains almost completely unblocked by [menthol] <1 mm. The data therefore preserve the concept that menthol exerts a “noncompetitive” block (presumably at the 9´ site in the transmembrane pore), and this block can be measured over a wide range of [ACh].

**Figure 8.**
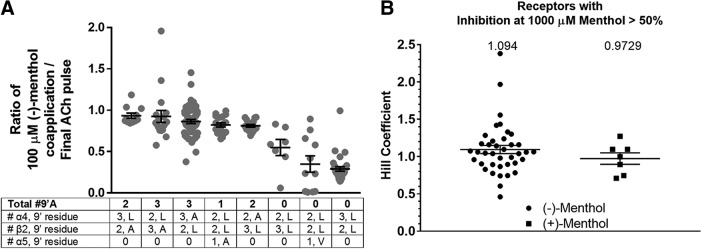
***A***, The ratio of currents elicited by an EC_50_ concentration of ACh and 100 µm menthol over the current elicited by ACh alone. Error bars represent the SEM; *n* = 7-33 oocytes. ***B***, Average Hill coefficients for wild-type and all mutant α4β2 mutants tested (*p* = 0.07). Error bars represent the SD; *n* = 7-39 mutants

Next, we determined the minimum number of 9´ mutations, within an nAChR pentamer, required to perturb the activity of menthol. Because the L9´A mutation most drastically reduced menthol sensitivity, we varied the number of L9´A mutations within the receptor pentamer. Since nAChRs are pseudosymmetric with fivefold symmetry, we can make up to five 9´A mutations. We made mutants with one, two, and three 9´A mutations since (α4[L9´A])_3_(β2)_2_ proved to be unaffected by menthol (Fig. 8*A*). We were also interested in determining whether this was a subunit-dependent effect, so we incorporated 9´A mutations in either β2 or α5 nAChR subunits. In all the mutants tested, as long as there was at least one 9´A mutation in α4, α5, or β2 nAChR subunits, inhibition by 100 µm (−)-menthol was significantly reduced (unpaired *t* test, *p* = 0.0001; Fig. 8*A*). The reduction in the ability of menthol to inhibit nAChRs that harbor 9´A mutations suggests that menthol binds to this site in TM2.

### One bound menthol molecule is sufficient for α4β2 inhibition

Hill coefficients, which describe the overall steepness of the concentration–response relation, are often used to gain an understanding of ligand–receptor binding stoichiometry (Prinz, 2010). We investigated the minimum number of bound menthol molecules necessary for channel inhibition. To test this, we calculated the Hill coefficients for both stereoisomers of menthol on all of the mutants tested (Fig. 8*B*). Since the average Hill coefficient is not significantly different from unity for either (−)-menthol or (+)-menthol, we concluded that the presence of a single bound menthol molecule is sufficient for nAChR inhibition.

### The activity of (+)-menthol and (−)-menthol depends on the end-to-end length of the 9´ residue

**Figure 9.**
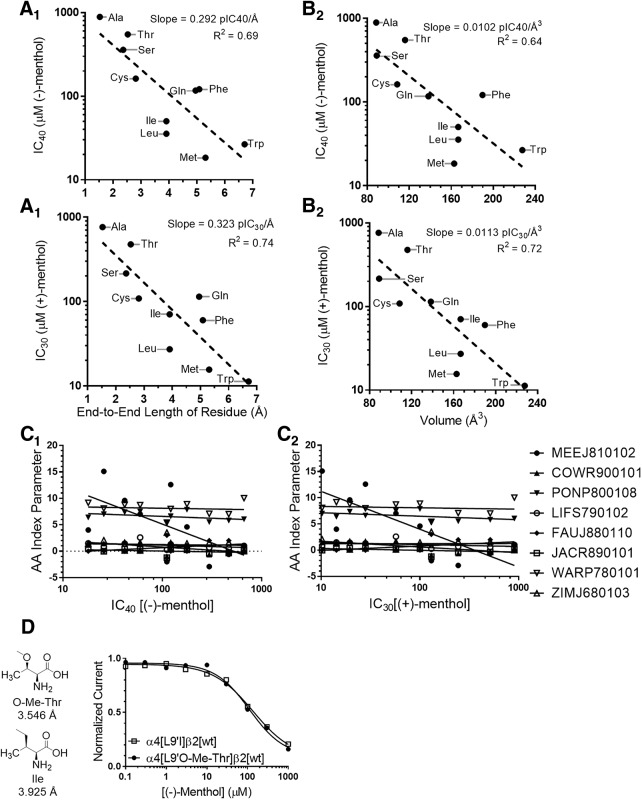
Potency of menthol depends on L9´ residue size. ***A_1_***–***B_2_***, Plot of IC_40_ for (−)-menthol versus residue length (***A_1_***) or residue volume (***B_1_***) and plot of IC_30_ for (+)-menthol versus residue length (***A_2_***) or residue volume (***B_2_***). ***C_1_***, Fitting the IC_40_ values for (−)-menthol against the reduced AA index. ***C_2_***, Fitting the IC_30_ values for (+)-menthol against the reduced AA index. *R* ^2^ values in ***C_1_*** and ***C_2_*** for each parameter are indicated in Figure 9-1 and Figure 9-2. ***D***, Concentration–response relationship for (−)-menthol on (α4[L9´I])_3_(β2)_2_ and (α4[L9´O-Me-Thr])_3_(β2)_2_ nAChRs. Structures of the two amino acids are shown to the left along with their end-to-end length; *n* = 7–18 oocytes.

Which properties of the side chain at 9´ are important for the inhibitory activity of menthol on α4β2 nAChRs? We examined the IC_40_ or IC_30_ values for (−)-menthol and (+)-menthol, respectively, against the 9´ residue volume, end-to-end length, and other parameters in the reduced amino acid index (AA index) (Fig. 9, Fig. 9-1, and Fig. 9-2; Zamyatnin, 1972; Kibinge et al., 2014). We calculated an additional parameter, end-to-end residue length, using a 6-31G* basis set Hartree–Fock calculation in SPARTAN ’14 (Wavefunction). We choose to use IC_40_ or IC_30_ so that the values calculated remained within the limits of our experimental data (Fig. 7*A1*,*A2*) because many of our dose–response studied failed to reach 100% inhibition at 1 mm menthol. We obtained a satisfactory relation when we compared the end-to-end length of residues with the IC_40_ of (−)-menthol; the correlation coefficient was 0.69 (Fig. 9*A1*). For (+)-menthol (IC_30_), we observed a correlation coefficient of 0.74 (Fig. 9*A2*). Thus, the potency of either stereoisomer of menthol exhibited a strong correlation with residue end-to-end length. We observed similar correlations when we compared the IC_40_ or IC_30_ of (−)-menthol and (+)-menthol, respectively, to residue volume (Fig. 9*B1*,*B2*).

10.1523/ENEURO.0465-18.2018.supplement Supplementary figure 9-1 R^2^ Values for correlation of IC_40_ for (−)-menthol vs. amino acid indices. Download Figure 9-1, DOCX file

10.1523/ENEURO.0465-18.2018.supplement Supplementary figure 9-2 R^2^ Values for correlation of IC_30_ for (+)-menthol vs. amino acid indices. Download Figure 9-2, DOCX file

Interestingly, previous data show that the EC_50_ of agonists depends strongly on polarity, a different property of the side chain at the 9´ position (Kearney et al., 1996; Kosolapov et al., 2000). To test whether polarity is a factor for menthol-induced inhibition, we incorporated O-methyl-threonine (O-Me-Thr) at the 9´ position. The end-to-end length of O-Me-Thr is just 0.41 Å longer than isoleucine (3.55 vs 3.14 Å), but because of the oxygen instead of a methylene unit in the side chain, O-Me-Thr is substantially more polar than isoleucine. If polarity at the 9´ position is important for menthol inhibition, we would expect to measure a meaningful difference in the concentration–response curve for menthol inhibition at these two mutants. We observed that the concentration–response relationships overlapped when comparing α4[L9´I]β2 to its O-Me-Thr variant (Fig. 9*C*). This suggests that menthol inhibition does not depend on the polarity of the 9´ side chain.

Additionally, we fitted the IC_40_ or IC_30_ values for (−)-menthol and (+)-menthol, respectively, against the reduced AA index (Kibinge et al., 2014). The reduced AA index is a set of eight indices describing the variability of amino acids based on experimental results. The reduced AA index is a subset of the AA index after the elimination of redundancies. We found that the end-to-end residue length and residue volume had more meaningful correlations than any parameter in the reduced AA index.

The two stereoisomers of menthol inhibit α7 nAChRs with similar IC_50_ values, but differences have been observed on the GABA_A_ receptors, biomembrane models, and mouse odorant receptors (Corvalán et al., 2009; Ashoor et al., 2013b; Takai and Touhara, 2015; Gusain et al., 2017). To further compare the potency of (−)-menthol and (+)-menthol on α4β2 nAChRs, we compared the percentage inhibition by 100 µm menthol along with the IC_50_ and pIC_30_ values on the 10 (α4[L9´X])_2_(β2)_3_ nAChRs and wild-type (α4)_3_(β2)_2_ nAChR (Fig. 7*C*,*D1*,*D2*). These scatterplots show either a comparison of the inhibition data (Fig. 7*B*) or comparison to a logarithmic transformation (Fig. 7*D1*,*D2*). We excluded the IC_50_ values for (α4[L9´A])_2_(β2)_3_ because we failed to observe inhibition of 50% of the control response. The data extend the experiments on wild-type nAChRs (Fig. 4), which showed modestly less inhibition by the (+) stereoisomer: (+)-menthol was on average 25% less potent than (−)-menthol (Fig. 7*D1*,*D2*).

## Discussion

Throughout the history of menthol-flavored tobacco products, various methods have been used to obtain the menthol used in cigarette production. Isolations from natural sources (mint leaves and oils) primarily produce (−)-menthol; most synthetic methods produce a racemic mixture of both (+)-menthol and (−)-menthol. The exception is the asymmetric synthesis developed by Ohkuma et al. (2000). We found that (+)-menthol and (−)-menthol exhibit different effects on α4β2 nAChR upregulation (Fig. 1). We also observed differences among the menthol stereoisomers in their ability to alter dopamine neuron firing frequency and excitability (Figs. 2, 3). Our previous investigations into the actions menthol used (±)-menthol (Henderson et al., 2016, 2017). Previous reports observed that (±)-menthol by itself upregulated nAChRs, altered dopamine neuron firing, enhanced the upregulation of nicotine-induced nAChR upregulation, and enhanced nicotine reward-related behavior. Because the present investigation shows that (+)-menthol has no effect on nAChR upregulation and dopamine neuron excitability, we conclude that previous observations of long-term menthol exposure and its effect on nAChR upregulation and dopamine neuron excitability were likely caused primarily by (−)-menthol only.

Because of the contrast between the high concentration of menthol required for acute inhibition of nAChRs (>30 µm) and the submicromolar effects on upregulation and dopamine neuron firing (≤ 500 nm), it is likely that menthol acts via multiple targets. The simulations and data in this study strongly suggest that the acute inhibitory effect of menthol is mediated through its direct interaction with nAChRs at the 9´ leucine site within the TM2 region. In previous electrophysiological experiments, well characterized nAChR blockers have permanent positive charges (e.g., QX-222) or can be protonated (e.g., mecamylamine) and bind near this site (at the 6´ and 10´ positions) by approaching through the open pore (Charnet et al., 1990; Lester, 1992; Papke et al., 2013). However, other experiments suggest that uncharged, membrane-permeant blockers, such as deprotonated procaine, can bind to the 6´ and 10´ residues in closed channels by approaching through the membrane or through the receptor protein (Adams, 1977; Lester, 1992). The simulations in this study reinforce the concept that menthol approaches the 9´ site in closed α4β2 nAChR channels by approaching from the protein. Our simulations are uninformative about a possible approach from the membrane phase, because the parameters were adjusted to minimize accumulation in the membrane phase (see Materials and Methods).

The experiments also show that (−)-menthol is only slightly (∼25%) more effective than (+)-menthol at inhibiting WT receptors (Fig. 4). This difference would be neglected if the dataset contained only WT receptors, but it is consistent and significant when assessed over a series of 9´ mutations that encompass a nearly 1000-fold range of sensitivities to menthol block (Fig. 7*D1*,*D2*). S(+)mecamylamine is a slightly more potent blocker than R(−)mecamylamine over a series of nAChR subtypes (Papke et al., 2013). These differences between stereoisomers as nAChR blockers are markedly less than the threefold to fourfold greater potency of (−)-menthol versus (+)-menthol at TRPM8 channels (Sherkheli et al., 2010).

The submicromolar chronic effects of menthol may result from a non-nAChR target. In addition to its actions on nAChRs, menthol acts as a positive modulator on GABA_A_ and glycine receptors and a noncompetitive antagonist of 5-HT_3_ receptors (Hall et al., 2004; Ashoor et al., 2013a). Menthol has well studied actions on TRP family proteins, especially TRPM8 and TRPA1 (Oz et al., 2017). Menthol also acts on other targets (Oz et al., 2017). Therefore, while menthol has acute actions on nAChRs (noncompetitive inhibition) through an allosteric interaction at the 9´ site, its effects on nAChR upregulation and dopamine neuron excitability may be caused by another protein target. Previous investigators suggested that menthol acts as a chemical chaperone (Henderson et al., 2016), in part because menthol enhanced the ER export of nAChRs. This designation has both analogies and differences with the pharmacological chaperoning of nAChRs by nicotine (Kuryatov et al., 2005; Srinivasan et al., 2011). Menthol may indeed be a chemical chaperone for nAChRs; but, given its ability to bind to many proteins of interest, we must consider that the enhancement of ER export by menthol may arise from its actions on a non-nAChR target related to anterograde trafficking of proteins or membranes.

As discussed earlier, menthol enhances nicotine reward (Henderson et al., 2017) and nicotine reinforcement (Wang et al., 2014; Biswas et al., 2016) in rodent models and produces poorer cessation rates in human smokers (Food and Drug Administration, 2012). Although the predominant form present in current tobacco products is (−)-menthol, the composition of menthol used in many tobacco products is presently neither disclosed nor regulated. For this reason, it is important to understand how both (+)-menthol and (−)-menthol alter the nAChRs on dopamine neurons that are involved in nicotine reward and reinforcement. This is especially important given that the ENDS market will become subject to systematic regulatory control by the Food and Drug Administration. Our data suggest that menthol exhibits a stereospecific effect only with low-dose, long-term exposure. We hypothesize that of the two primary menthol stereoisomers, only (−)-menthol plays a role in enhancing nicotine reward through nAChRs on dopamine neurons. We do not yet suggest that (+)-menthol lacks any role in nicotine reward. GABA neurons in the VTA and SNr play an important role in nicotine reward as their disinhibition plays a critical role in the ability of nicotine to drive enhancements, dopamine neuron excitability, and dopamine release (Mansvelder et al., 2002; Nashmi et al., 2007). Menthol does act as a positive allosteric modulator of GABA_A_ receptors and (+)-menthol is more potent than (−)-menthol on these receptors (Hall et al., 2004; Corvalán et al., 2009). Given that stereospecific effects of menthol have been observed with nAChRs and GABA receptors (both important targets for nicotine reward), there is a continued need to understand their distinct pharmacology and how it alters the actions of nicotine on midbrain neurons.

Given the reduced effect (+)-menthol exhibits on dopamine neurons, would public health benefit from availability of combustible products in which (+)-menthol replaces (−)-menthol? Because (+)-menthol is also less effective at activating TRPM8, (+)-mentholated products might also less effectively provide the characteristic cooling sensation. Smokers might therefore continue to prefer tobacco flavored with (−)-menthol (Sherkheli et al., 2010). We also have no information about stereospecificity of TRPA1 activation by either menthol or nicotine (Talavera et al., 2009). This report, and others (Alsharari et al., 2015; Biswas et al., 2016; Fan et al., 2016; Henderson et al., 2016, 2017), provide evidence both that (−)-menthol plays a role in enhancing the addiction to nicotine, and also that simply substituting (+)-menthol for (−)-menthol in combustible tobacco products may not be the correct strategy for harm reduction.

## Synthesis

Reviewing Editor: Karen Szumlinski, University of California at Santa Barbara Decisions are customarily a result of the Reviewing Editor and the peer reviewers coming together and discussing their recommendations until a consensus is reached. When revisions are invited, a fact-based synthesis statement explaining their decision and outlining what is needed to prepare a revision will be listed below. Note: If this manuscript was transferred from JNeurosci and a decision was made to accept the manuscript without peer review, a brief statement to this effect will instead be what is listed below.

I have read this transferred manuscript and found the study to be well-executed and the report is well-written. The data provide new information pertaining to the effects of menthol on the brain of relevance to tobacco/nicotine addiction. I am happy to accept this report as is.
