# Supplementary material for: Menthol Stereoisomers Exhibit Different Effects on α4β2 nAChR Upregulation and Dopamine Neuron Spontaneous Firing
Source: eNeuro. 2019 Jan 4;5(6):ENEURO.0465-18.2018. doi: 10.1523/ENEURO.0465-18.2018 (PMC6325563; doi:10.1523/ENEURO.0465-18.2018)
Supplement: Supplementary figure 7-1 — p-values for (+)-menthol and (−)-menthol comparison between α4[L9‵X]β2[wt] mutants. Download Figure 7-1, DOCX file [file sup_enu-eN-NWR-0465-18-s01.docx]

**Extended Data**

| **Figure 7-1. p-values for (+)-menthol and (−)-menthol comparison between α4[L9’X]β2[wt] mutants** | |
| --- | --- |
| **Receptor** | **p-value (from paired, two-tailed t-test)** |
| (α4[L9'T])_2_(β2[wt])_3_ | 2.21 x 10^-4^ |
| (α4[L9'A])_2_(β2[wt])_3_ | 3.79 x 10^-7^ |
| (α4[L9'S])_2_(β2[wt])_3_ | 8.07 x 10^-5^ |
| (α4[L9'Q])_2_(β2[wt])_3_ | 1.29 x 10^-3^ |
| (α4[L9'C])_2_(β2[wt])_3_ | 2.19 x 10^-3^ |
| (α4[L9'F])_2_(β2[wt])_3_ | 0.793 |
| (α4[L9'I])_2_(β2[wt])_3_ | 7.14 x 10^-4^ |
| (α4[wt])_2_(β2[wt])_3_ | 0.957 |
| (α4[L9'M])_2_(β2[wt])_3_ | 5.70 x 10^-13^ |
| (α4[L9'W])_2_(β2[wt])_3_ | 0.411 |
| (α4[wt])_3_(β2[wt])_2_ | 1.00 x 10^-15^ |
