# Supplementary material for: Menthol Stereoisomers Exhibit Different Effects on α4β2 nAChR Upregulation and Dopamine Neuron Spontaneous Firing
Source: eNeuro. 2019 Jan 4;5(6):ENEURO.0465-18.2018. doi: 10.1523/ENEURO.0465-18.2018 (PMC6325563; doi:10.1523/ENEURO.0465-18.2018)
Supplement: Supplementary figure 9-1 — R2 Values for correlation of IC40 for (−)-menthol vs. amino acid indices. Download Figure 9-1, DOCX file [file sup_enu-eN-NWR-0465-18-s02.docx]

| **Figure 9-1. R^2^ Values for correlation of IC_40_ for (−)-menthol vs. amino acid indices** | |
| --- | --- |
| **Index** | **R^2^** |
| End-to-End length | 0.69 |
| Volume | 0.64 |
| MEEJ810102 | 0.37 |
| COWR900101 | 0.35 |
| PONP800108 | 0.19 |
| LIFS790102 | 0.19 |
| FAUJ880110 | 0.15 |
| JACR890101 | 0.094 |
| WARP78010 | 0.014 |
| ZIMJ680103 | 0.0066 |
